# Compliance and Impact of a 5-Min Seated Rest Protocol on Home Blood Pressure Monitoring in Postpartum Women

**DOI:** 10.1093/ajh/hpaf152

**Published:** 2025-08-13

**Authors:** Jae-Myung Kim, Bethany Barone Gibbs, Kara M Whitaker

**Affiliations:** Department of Health & Human Physiology, University of Iowa, Iowa City, Iowa, USA; Department of Epidemiology and Biostatistics, West Virginia University, Morgantown, West Virginia, USA; Department of Health & Human Physiology, University of Iowa, Iowa City, Iowa, USA

**Keywords:** accelerometer, blood pressure, blood pressure measurement recommendations, home blood pressure monitoring, hypertension, postpartum

## Abstract

**BACKGROUND:**

Home blood pressure monitoring (HBPM) is an effective method for diagnosing and managing postpartum hypertension, a condition associated with increased health risks. A 5-min seated rest before home blood pressure (BP) measurement is recommended; however, compliance to this recommendation and its impact on HBPM reading in postpartum women is unknown.

**METHODS:**

A subset of participants enrolled in a pregnancy cohort were followed at 3 and 6 months postpartum. At each assessment, participants completed HBPM for seven days with an oscillometric device and concurrently wore an accelerometer on their thigh to assess postures. Mixed-effects models and intraclass correlation coefficients were utilized to analyze BP differences and measurement reliability between 5-min rest compliant and noncompliant readings, respectively.

**RESULTS:**

A total of 45 participants (mean age: 30.5 years) provided HBPM data at 3 and/or 6 months postpartum, with 90.2% of requested BP measures taken. Approximately 33% of readings adhered to the 5-min rest protocol. Compliant readings averaged lower systolic and diastolic BP values than noncompliant readings (SBP: 105.9 mmHg vs. 107.1 mmHg; DBP: 72.6 mmHg vs. 73.2 mmHg), but differences were not clinically relevant. Compliant DBP ICCs fell within the good reliability range (ICCs: 0.785–0.817), while other ICCs indicated moderate reliability.

**CONCLUSIONS:**

Despite low compliance with 5 mins of seated rest prior to HBPM, the minimal impact on BP values suggests HBPM remains a useful monitoring strategy in postpartum women, even if the premeasurement rest is not always possible. Future research could evaluate whether shorter premeasurement rest recommendations produce similar findings.

## Graphical Abstract

**Graphical Abstract:**
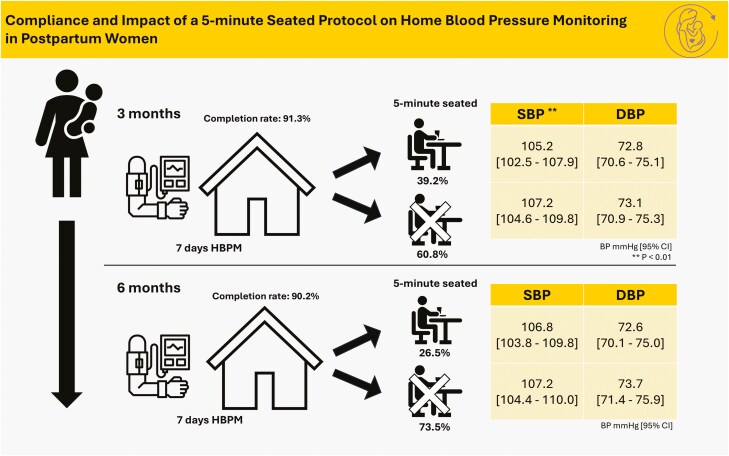

High blood pressure (BP) is a primary risk factor for cardiovascular disease (CVD) and is commonly observed in the postpartum period, particularly among women with hypertensive disorders of pregnancy (HDP). Approximately half of women with HDP and 10%–20% with normotensive pregnancies develop postpartum hypertension.^1–3^ Given the high incidence of postpartum hypertension and associated CVD risks, accurate and consistent assessment of BP following delivery through remote BP monitoring is an essential clinical strategy to allow for early intervention and improved outcomes.^2,4^

Home blood pressure monitoring (HBPM) has emerged as a valuable tool in managing hypertension in mid-late postpartum.^4^ It allows frequent measurements in a familiar environment, providing a more accurate representation of typical BP. HBPM also empowers women to actively manage their health, leading to better adherence and outcomes.^5^ Additionally, HBPM helps identify masked hypertension, which is characterized by normal BP readings in a clinical setting but elevated readings at home and is estimated to affect 10%–20% of postpartum women.^6–8^ Similarly, white coat hypertension, where BP readings are elevated in a clinical setting but normal at home, affects approximately 10% of postpartum women and could lead to overdiagnosis of hypertension without additional HBPM.^8^ Therefore, ensuring accurate diagnosis of hypertension using remote BP monitoring methods (e.g., HBPM) is crucial for effective BP management in postpartum women.

American Heart Association guidelines for measuring BP recommend that individuals sit quietly for at least 5 min before taking a BP measurement to ensure the accuracy of readings.^9^ An insufficient rest period is a key factor contributing to inaccurate in-clinic BP measures,^10^ as the stimulus of arriving at the clinic could affect readings.^11^ However, recent randomized trials and quality improvement studies have challenged the necessity of a 5-min rest in the context of automated office blood pressure (AOBP) and ambulatory blood pressure monitoring (ABPM). In AOBP measurement, the differences between 0-min or 2-min rest periods compared to 5-min rest periods were less than 5 mmHg.^12,13^ Furthermore, AOBP readings closely aligned with day-long ABPM readings regardless of the rest period.^14^ These findings suggest that a 5-min rest may have a minimal impact on BP readings, yet similar evaluation of shorter rest periods during HBPM are limited.

Despite the recommendation of a 5-min rest prior to measurement for HBPM,^15^ adherence rates to this rest and the impact of noncompliance on HBPM readings remain to be investigated. This is of particular interest for BP management in a postpartum population at high risk for developing hypertension, who may have unique barriers to rest compliance. Therefore, this study aimed to evaluate compliance with a 5-min seated rest prior to HBPM and its impact on BP readings among postpartum women at 3 and 6 months using objectively measured sitting posture.

## METHODS

This secondary analysis utilized data collected by the Postpartum 24/7 pilot cohort study, which aimed to examine the associations between 24-h activity patterns and cardiovascular health across the postpartum period.

### Participants

The Postpartum 24/7 study recruited a subsample of 50 participants from the Pregnancy 24/7 cohort study^16^ (NCT04749849) at the University of Iowa (*n* = 30) and West Virginia University (*n* = 20). Women were excluded from the Pregnancy 24/7 study if they (i) used BP- or glucose-lowering medications; (ii) were unable to walk half a mile or climb two flights of stairs; or (iii) had serious medical conditions such as severe CVD, cardiomyopathy, or heart failure. In addition, women were excluded from the Postpartum 24/7 study if they were newly pregnant. A total of 50 and 49 participants completed the 3- and 6-month postpartum study visits and were eligible for this study.

### Summary of procedures

At 3 and 6 months postpartum, participants attended virtual study visits. Five days prior to each visit, a research assessment package was sent to participants, which included a BP monitor, two activity monitors, medical dressings, a sleep diary, and instructions for the usage of the BP and activity monitors. During the study visit, participants provided their medical history via questionnaires, took practice BP measurements, and placed the activity monitors. The research assistant instructed participants on the BP measurement protocol according to HBPM measurement recommendations,^15^ emphasizing the 5-min seated rest before the first measurement.

Following the study visit, participants engaged in HBPM for seven consecutive days while wearing the activity monitors. At the end of the monitoring period, participants were instructed to keep the BP monitor for use in subsequent assessments and return the activity monitors and sleep diary using a prepaid postal envelope.

### BP measurement

BP was assessed using an automatic oscillometric upper-arm BP monitor (BP7250, Omron Healthcare, Inc., Kyoto, Japan), which was validated for clinical accuracy according to the universal validation protocol.^17^ Participants were instructed to synchronize their Omron monitor with an associated smartphone application (OMRON connect US/CAN/EMEA, Omron Healthcare, Inc., Hoffman Estates, IL, USA), to facilitate the transfer of their systolic blood pressure (SBP) and diastolic blood pressure (DBP) data. To prevent data loss due to device malfunction, participants also manually recorded their BP readings in a sleep diary.

Participants were asked to take four BP readings each day: two in the morning upon waking and two in the evening before bedtime. Each reading was to be taken following 5 min of seated rest, with 1 min rest between readings. On average, 28 BP readings (2 readings × morning and night × 7 days) were collected per person. Following the completion of the monitoring, participants exported their BP data from the OMRON connect and sent it to the research team via email. Only measures taken on days 2–8 of monitoring were used for analyses because those taken on the first day were considered practice.

### Posture measurement

The activPAL3 micro (PAL Technologies Ltd, Glasgow, Scotland), a thigh-worn accelerometer, was employed to assess sitting posture prior to each reading. The device classifies between sitting, standing, stepping, and lying postures based on changes in thigh inclination, estimated via acceleration signals. Previous validation studies have demonstrated its precision and reliability in measuring free-living sedentary time.^18,19^

The activPAL was prepackaged by the research team using a finger cot and retention tape. Participants self-applied the activity monitor to the anterior right thigh using transparent medical dressing (Tegaderm, 3M Corporation, Maplewood, MN, USA) and wore it continuously (24 h a day) throughout the monitoring period. Data was integrated into 60-second epochs using the CREA (version 1.3) algorithm within proprietary PALanalysis software (version 8.11, PAL Technologies Ltd, Glasgow, Scotland) for analysis.

### The 5-min resting compliance evaluation

We retrieved activPAL data for 10 min prior to each BP measurement time. We then aggregated the time spent during the pre-BP measurement intervals in different postures, including sitting, standing, stepping, and lying. In accordance with the BP measurement guidelines, which recommend resting quietly in a seated position, we considered a sitting posture to be compliant.^9^ BP readings were censored if they were (i) recorded during sleep time, (ii) associated with continuous lying posture, and (iii) had invalid time data. Following these procedures, participants with less than three days of BP readings (<12 readings)^20^ were excluded from analysis.

Compliance with the 5-min seated rest recommendation was assessed based on the first BP reading of two consecutive readings, resulting in up to 14 weekly evaluations per participant. Due to the differing minimum units of the Omron monitor (minutes) and activPAL (seconds), we evaluated compliance as at least 240 s (4 min) of continuous sitting prior to the recorded minute.

### Statistical analysis

Participant characteristics were presented using means and standard deviation or frequencies and percentages. The proportion of time spent in each posture during the 5 min prior to BP measurement was calculated for both compliant and noncompliant readings. The 5-min rest compliance rate was calculated as the ratio of BP readings with completed rest to the total number of reported readings per participant.

To assess the impact of a 5-min rest on BP, we compared SBP and DBP between compliant and noncompliant readings using a linear mixed-effects model. Participants and study visits were designated as random effects, and 5-min rest compliance as a fixed effect. Additionally, two submodels were created to analyze the impact at 3 and 6 months postpartum separately. Residuals were assessed for normality and homoscedasticity through visual inspection, and these assumptions were confirmed. An unstructured covariance matrix was selected due to its lower Akaike Information Criterion value. The estimated marginal means with standard errors and 95% confidence intervals (CI) were estimated to evaluate the difference between compliant and noncompliant BP readings.

Additionally, intraclass correlation coefficients (ICC) were applied to evaluate the consistency of HBPM readings. ICCs were calculated as the ratio of within-participant variance to total residual variance, using separate linear mixed-effects models for 5-min rest compliant and noncompliant readings. The 95% CI of ICC was calculated using a bootstrapping procedure with 1,000 resamples.

We used R statistical software version 4.4.1 for data manipulation and statistical analysis.^21^ The linear mixed-effects model was built via the “*nlme*” R package.^22^ The statistical significance was tested at an alpha level of 0.05.

The research protocol herein was approved by the Institutional Review Board of the University of Iowa (202002630) with reliance from West Virginia University. All participants in this study gave their written informed consent.

## RESULTS

Of the 50 study participants, 45 completed HBPM during either the 3-month (*n* = 41) or 6-month (*n* = 34) postpartum assessment.

### Sample characteristics

As seen in **Table 1**, study participants had a mean age of 30.5 ± 4.5 years, with a prepregnancy body mass index (BMI) of 27.3 ± 6.4 kg/m^2^. A total of 15.6% experienced gestational hypertension and 4.4% had preeclampsia in their recent pregnancy. Based on in-office BP measurements within 6 weeks postpartum, mean SBP and DBP values were 119.2 ± 13.8 mmHg and 74.7 ± 9.5 mmHg, respectively, and 41.9% of participants had elevated or stage 1 hypertension.

**Table 1.** Characteristics of postpartum 24/7 pilot study participants

Characteristics	*N* = 45
	Mean ± SD or *n* (%)
Age (years)	30.5 ± 4.5
Prepregnancy BMI (kg/m^2^)	27.3 ± 6.4
Race/Ethnicity	
White	38 (84.4)
Asian	4 (8.9)
Multiracial	3 (6.7)
Marital status	
Married	36 (80.0)
A marriage-like relationship	6 (13.3)
Single/not married	3 (6.7)
Insurance	
Medicaid	6 (13.3)
Private	39 (86.7)
History of HDP	
Gestational Hypertension	7 (15.6)
Preeclampsia	2 (4.4)
Postpartum BP (mmHg)	
Systolic	119.2 ± 13.8
Diastolic	74.7 ± 9.5
Postpartum hypertension	
Hypertension	15 (34.9)
Elevated	3 (7.0)
Normal	25 (58.1)

Postpartum hypertension was diagnosed based on a clinical visit at 6 weeks postpartum.

Abbreviations: BMI: body mass index; BP: blood pressure; HDP: hypertensive disorders of pregnancy.

### HBPM adherence and 5-min rest compliance

As seen in **Table 2**, on average participants provided 25.6 readings (91.3%) at 3 months postpartum and 25.3 readings (90.2%) at 6 months postpartum. The overall compliance rate for the 5-min rest was 33.4%. Specifically, 39.2% and 26.5% of readings were measured after a 5-min rest at 3 and 6 months postpartum, respectively.

**Table 2.** Characteristics of postpartum HBPM

	Overall	3 months (*N* = 41)	6 months (*N* = 34)
Number of readings, *n* (%)	25.4 (90.7)	25.6 (91.3)	25.3 (90.2)
Compliance rate, %	33.4	39.2	26.5
Blood pressure			
SBP, mmHg	106.8 ± 7.8	106.4 ± 5.6	107.2 ± 5.3
DBP, mmHg	73.2 ± 6.5	73.1 ± 4.1	73.3 ± 3.8
			
Elevated & higher, n (%)	–	5 (12.2)	5 (14.7)
Normal, *n* (%)	–	36 (87.8)	29 (85.3)

Compliance rate = 5-min rest compliant readings/total readings.

**Figure 1.**
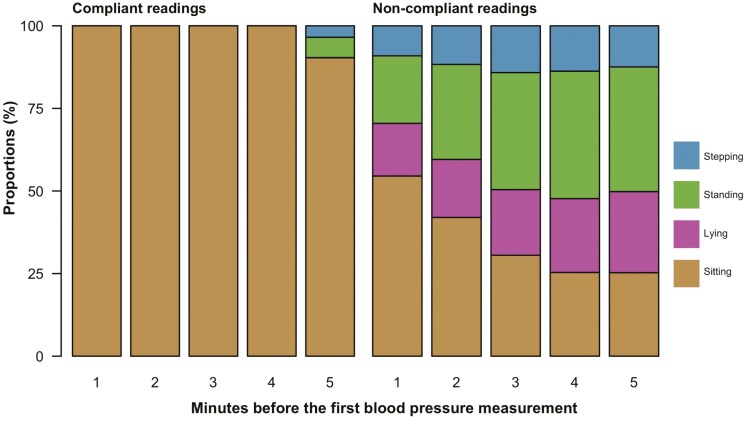
The proportion of sitting, lying, standing, and stepping at each minute before the first BP measurement. The bar chart of compliant readings illustrates a consistent 100% sitting posture in the 4 min leading up to the first BP measurement. In contrast, noncompliant readings show an average of less than 50% sitting posture, equivalent to 30 s, throughout the 5 min.

**Figure 1** illustrates the proportion of sitting, lying, standing, and stepping postures over 5 min prior to the first BP measurement. Sitting posture was predominant during the 5 min preceding the rest compliant BP measurements. On average, noncompliant measures were preceded by 35.5% (range: 25.3%–54.5%) sitting, 32.2% (20.4%–38.6%) standing, 20.1% (15.9%–24.5%) lying, and 12.2% (9.1%–14.2%) stepping.

### BP readings by 5-min rest compliance

As seen in **Table 3**, estimated mean SBP with 5-min rest compliance demonstrated a lower value compared to noncompliant readings across 3 and 6 months postpartum (105.9 ± 1.2 vs. 107.1 ± 1.2 mmHg, P = 0.018). At 3 months postpartum, estimated mean SBP with compliant readings was 2.06 mmHg lower than noncompliant readings (105.2 ± 1.3 vs. 107.2 ± 1.3 mmHg, *P* < 0.01). At 6 months postpartum, however, we found no statistically significant difference between compliant and noncompliant SBP (106.8 ± 1.5 vs. 107.2 ± 1.4 mmHg, *P* = 0.626).

**Table 3.** The difference between compliant and noncompliant BP readings at 3 and 6 months postpartum

		EMM	SE	95% CI		Δ_C-NC_	*t*	P
SBP								
Overall	C	105.9	1.2	103.3	108.4	−1.230	−2.370	0.018
	NC	107.1	1.2	104.7	109.5			
3 m	C	105.2	1.3	102.5	107.9	−2.056	−2.743	0.006
	NC	107.2	1.3	104.6	109.8			
6 m	C	106.8	1.5	103.8	109.8	−0.358	−0.488	0.626
	NC	107.2	1.4	104.4	110.0			
DBP								
Overall	C	72.6	1.0	70.5	74.6	−0.637	−1.691	0.091
	NC	73.2	1.0	71.2	75.2			
3 m	C	72.8	1.1	70.6	75.1	−0.273	−0.510	0.610
	NC	73.1	1.1	70.9	75.3			
6 m	C	72.6	1.2	70.1	75.0	−1.100	−2.041	0.042
	NC	73.7	1.1	71.4	75.9			

Abbreviations: C, compliant BP readings; NC, noncompliant BP readings; EMM, estimated marginal means; SE, standard error.

DBP readings with 5-min rest compliance demonstrated similar estimated mean values compared to noncompliant readings across 3 and 6 months postpartum (72.6 ± 1.0 vs. 73.2 ± 1.0 mmHg, *P* = 0.091). There was no statistically significant difference between compliant and noncompliant readings at 3 months postpartum (*P* = 0.610). At 6 months postpartum, the compliant DBP was 2.04 mmHg lower than noncompliant readings (72.6 ± 1.2 vs. 73.7 ± 1.1 mmHg, *P* = 0.042).

### Consistency of BP by 5-min rest compliance

ICCs were calculated to assess the consistency of BP readings under compliant and noncompliant conditions, demonstrating higher ICCs with 5-min compliant readings overall. The ICCs for SBP were 0.670 (95% CI: 0.575, 0.763) for compliant readings and 0.598 (95% CI: 0.531, 0.661) for noncompliant readings. For DBP, the overall ICCs were 0.785 (95% CI: 0.683, 0.844) for compliant readings and 0.604 (95% CI: 0.542, 0.657) for noncompliant readings. Separate ICC values for 3 and 6 months postpartum are presented in **Table 4**.

**Table 4.** The ICC of compliant and noncompliant BP readings at 3 and 6 months postpartum

		Compliant readings			Noncompliant readings		
		ICC	95% CI		ICC	95% CI	
SBP	Overall	0.670	0.575	0.763	0.598	0.531	0.661
	3 m	0.698	0.559	0.763	0.685	0.594	0.708
	6 m	0.609	0.407	0.652	0.704	0.625	0.716
DBP	Overall	0.785	0.683	0.844	0.604	0.542	0.657
	3 m	0.817	0.735	0.874	0.720	0.636	0.736
	6 m	0.778	0.673	0.802	0.735	0.659	0.756

## DISCUSSION

This study investigated the compliance and impact of a 5-min rest on HBPM in postpartum women at 3 and 6 months. The participants demonstrated 90.7% adherence to obtain HBPM readings (2 measures × twice per day × 7 days) throughout the follow-up. Despite completing most HBPM readings, less than half of the BP readings (33.4%) followed the 5-min rest protocol before the first measurement. Although compliant and noncompliant readings showed marginal or statistically significant differences, up to 2 mmHg, this small magnitude suggests limited clinical relevance. A prior meta-analysis indicates that at least a 5-mmHg reduction in SBP is necessary to reduce the risk of major cardiovascular events.^23^ Furthermore, ICCs demonstrated moderate to good reliability for both SBP and DBP readings in both compliant and noncompliant conditions.

Several guidelines and statements recommended a self BP monitoring schedule optimally comprising two measurements each in the morning and night over 7 days, for a minimum of 3 days.^15,24,25^ In addition, a previous HBPM reliability study suggested that a protocol of one measurement each in the morning and night for three consecutive days provides a reliable estimate of HBPM.^26^ Based on these recommendations, 6 to 28 measurements per week are generally considered sufficient for obtaining a reliable estimate of BP. Herein, our participants demonstrated a high level of HBPM protocol adherence, on average completing 25.4 out of 28 scheduled measurements over seven days. Consistent with our findings, a previous case–control study revealed that women 1 to 4 years postpartum with a history of preeclampsia reported 13 out of 14 scheduled HBPM measurements.^7^ This high level of adherence suggests that the recommended HBPM schedule is acceptable for postpartum women, supporting its feasibility for risk management in this population.

Despite the high adherence to obtain the HBPM readings, our study participants demonstrated low compliance with the 5-min rest protocol. While direct comparisons of the 5-min rest compliance are limited by the paucity of prior research on HBPM, the impact of the 5-min rest on BP measurement has been discussed. A previous meta-analysis introduced insufficient rest as the source of inaccurate resting BP measurement in a clinic setting, with patients having significantly higher SBP and DBP if they did not rest for sufficient times.^10^ Furthermore, research in AOBP indicated that SBP and DBP were approximately 7 and 4 mmHg higher when measurements were taken without continuous seated posture lasting longer than 5 min.^27^ These findings highlight the importance of extended resting time for accurate BP measurements.

On the other hand, and comparable to the small differences observed in our study, a previous randomized clinical trial including middle-aged adults indicated a minimal impact of the 5-min rest on BP measurement. Compared to the 5-min rest, SBP differences were −1.7 mmHg (95% CI: −2.8, −0.6) for the 2-min rest and 0.2 mmHg (95% CI: −0.8, −1.2) for no rest. DBP differences remained less than 1.1 mmHg for both 2 min and no rest.^13^ Another previous randomized controlled trial investigating rest duration on AOBP observed lower mean SBP and DBP in the 5-min rest group compared to the 0-min rest group (SBP: 138.2 mmHg vs. 141.2 mmHg; DBP: 81.7 mmHg vs. 83.1 mmHg); again determining that differences did not meet clinical significance.^12^ Although the 5-min rest compliant readings in the current study demonstrated slightly improved ICCs, suggesting better consistency, most ICCs remained within the moderate range. Collectively, the evidence from previous studies and this research suggests that a 5-min rest period remains advisable for consistent and accurate BP estimates; however, failure to comply with this recommendation had only a minimal impact on HBPM values and consistency.

This study has several strengths regarding its methodology and target population. First, we utilized a BP monitor that has proven reliability, validity, and accessibility. Furthermore, we employed a smartphone application to download BP measurement data, supporting the objectiveness of the analysis. Second, our study objectively assessed compliance with the 5-min rest for the HBPM protocol, a factor that has been challenging to evaluate due to the difficulty of capturing free-living posture. We addressed this gap by evaluating the participants’ posture utilizing thigh-worn accelerometers. Lastly, we examined HBPM measurements in postpartum women, a population for whom BP management is critical for CVD risk management.^28–31^

Despite the aforementioned strengths, this study has several limitations. First, the study participants consisted of healthy women with and without a history of HDP, who presented with low SBP and DBP. This contrasts with a previous clinical study that reported HBPM tended to overestimate BP in postpartum women with elevated BP.^7^ Furthermore, the participants were predominantly married white women with private health insurance, lacking sociodemographic diversity. Therefore, the findings should be generalized with caution. Second, due to the observational cohort study design, some variables remained uncontrolled. Also, although we implemented screening procedures to identify misaligned and nonsensical data from both activPAL and Omron devices, the potential for minimal time gaps remains due to differences in their time measurement units. The device we employed to measure compliance classifies posture but does not assess other body position recommendations prior to BP measurement, such as having the back, feet, and arm supported. Lastly, we were unable to evaluate the sensitivity and specificity of rest-compliant vs. noncompliant readings since we lacked a comparison measure of true positives and negatives in this secondary analysis. Future clinical trials evaluating the current HBPM protocol should account for these limitations to further enhance the accuracy and reliability of their findings.

This study revealed that postpartum women were capable of completing a rigorous 7-day HBPM protocol, though compliance with a 5-min rest before BP measurement was less successful. Yet, noncompliance with the 5-min rest prior to BP measurement did not have a clinically significant impact on estimates of BP at 3 and 6 months postpartum. It is possible that shorter or no rest may be adequate for HBPM protocols for healthy postpartum women, though the effect of modifying instructions for HBPM should be specifically tested before HBPM measurement practices are altered. Overall, these data support the notion that HBPM after providing standardized instructions likely results in valid BP assessment, even if not all measures are truly resting.

## Data Availability

The data will be shared at reasonable request to the Physical activity & Women’s Health Lab, kara-whitaker@uiowa.edu.

## References

[1] Goel A, Maski MR, Bajracharya S, Wenger JB, Zhang D, Salahuddin S, Shahul SS, Thadhani R, Seely EW, Karumanchi SA, Rana S. Epidemiology and mechanisms of De Novo and persistent hypertension in the postpartum period. Circulation 2015; 132:1726–1733. doi: https://doi.org/10.1161/CIRCULATIONAHA.115.015721 26416810 PMC4816491

[2] Ackerman‐Banks CM, Grechukhina O, Spatz E, Lundsberg L, Chou J, Smith G, Greenberg VR, Reddy UM, Xu X, O’Bryan J, Smith S, Perley L, Lipkind HS. Seizing the window of opportunity within 1 year postpartum: early cardiovascular screening. J Am Heart Assoc 2022; 11:e024443. doi: https://doi.org/10.1161/JAHA.121.024443 35411781 PMC9238464

[3] Parker SE, Ajayi A, Yarrington CD. De Novo postpartum hypertension: incidence and risk factors at a safety-net hospital. Hypertension 2023; 80:279–287. doi: https://doi.org/10.1161/HYPERTENSIONAHA.122.1927536377603

[4] Lewey J, Beckie TM, Brown HL, Brown SD, Garovic VD, Khan SS, Miller EC, Sharma G, Mehta LS; American Heart Association Cardiovascular Disease and Stroke in Women and Underrepresented Populations Committee of the Council on Clinical Cardiology; Council on Cardiopulmonary, Critical Care, Perioperative and Resuscitation; and Council on Cardiovascular and Stroke Nursing. Opportunities in the postpartum period to reduce cardiovascular disease risk after adverse pregnancy outcomes: a scientific statement from the American Heart Association. Circulation 2024; 149:e330–e346. doi: https://doi.org/10.1161/CIR.0000000000001212 38346104 PMC11185178

[5] Hinton L, Tucker KL, Greenfield SM, Hodgkinson JA, Mackillop L, McCourt C, Carver T, Crawford C, Glogowska M, Locock L, Selwood M, Taylor KS, McManus RJ. Blood pressure self-monitoring in pregnancy (BuMP) feasibility study; a qualitative analysis of women’s experiences of self-monitoring. BMC Pregnancy Childbirth 2017; 17:427. doi: https://doi.org/10.1186/s12884-017-1592-1 29258469 PMC5735874

[6] Ditisheim A, Wuerzner G, Ponte B, Vial Y, Irion O, Burnier M, Boulvain M, Pechère-Bertschi A. Prevalence of hypertensive phenotypes after preeclampsia. Hypertension 2018; 71:103–109. doi: https://doi.org/10.1161/HYPERTENSIONAHA.117.09799 29133363

[7] Nuckols VR, Stroud AK, Armstrong MK, Brandt DS, Santillan MK, Santillan DA, Pierce GL. Postpartum ambulatory and home blood pressure monitoring in women with history of preeclampsia: diagnostic agreement and detection of masked hypertension. Pregnancy Hypertension 2022; 29:23–29. doi: https://doi.org/10.1016/j.preghy.2022.05.003 35671544 PMC9645805

[8] Espeche WG, Salazar MR. Ambulatory blood pressure monitoring for diagnosis and management of hypertension in pregnant women. Diagnostics (Basel, Switzerland) 2023; 13:1457. doi: https://doi.org/10.3390/diagnostics13081457 37189558 PMC10137869

[9] Muntner P, Shimbo D, Carey RM, Charleston JB, Gaillard T, Misra S, Myers MG, Ogedegbe G, Schwartz JE, Townsend RR, Urbina EM, Viera AJ, White WB, Wright JT. Measurement of blood pressure in humans: a scientific statement from the American Heart Association. Hypertension 2019; 73:e35–e66. doi: https://doi.org/10.1161/HYP.0000000000000087 30827125 PMC11409525

[10] Kallioinen N, Hill A, Horswill MS, Ward HE, Watson MO. Sources of inaccuracy in the measurement of adult patients’ resting blood pressure in clinical settings: a systematic review. J Hypertens 2017; 35:421–441. doi: https://doi.org/10.1097/HJH.0000000000001197 27977471 PMC5278896

[11] Einstadter D, Bolen SD, Misak JE, Bar-Shain DS, Cebul RD. Association of repeated measurements with blood pressure control in primary care. JAMA Internal Med 2018; 178:858–860. doi: https://doi.org/10.1001/jamainternmed.2018.0315 29710186 PMC6061958

[12] Tobe SW, Dubrofsky L, Nasser DI, Rajasingham R, Myers MG. Randomized controlled trial comparing automated office blood pressure readings after zero or five minutes of rest. Hypertension 2021; 78:353–359. doi: https://doi.org/10.1161/HYPERTENSIONAHA.121.17319 34176286

[13] Brady TM, Charleston J, Ishigami J, Miller ER, Matsushita K, Appel LJ. Effects of different rest period durations prior to blood pressure measurement: the best rest trial. Hypertension 2021; 78:1511–1519. doi: https://doi.org/10.1161/HYPERTENSIONAHA.121.17496 34601959

[14] Lynn-Green EE, Cluett JL, Turkson-Ocran RAN, Mukamal KJ, Li JX, Juraschek SP. Clinical impact of 3- vs. 5-minute delay and 30- vs. 60-second intervals on unattended automated office blood pressure measurements. Am J Hypertens 2025; 38:168–177. doi: https://doi.org/10.1093/ajh/hpae135 39387134 PMC11833244

[15] Shimbo D, Artinian NT, Basile JN, Krakoff LR, Margolis KL, Rakotz MK, Wozniak G; American Heart Association and the American Medical Association. Self-measured blood pressure monitoring at home: a joint policy statement from the American Heart Association and American Medical Association. Circulation 2020; 142:e42–e63. doi: https://doi.org/10.1161/CIR.0000000000000803 32567342

[16] Whitaker KM, Jones MA, Smith K, Catov J, Feghali M, Kline CE, Santillan M, Santillan D, Zimmerman B, Gibbs BB. Study design and protocol of the multisite pregnancy 24/7 cohort study. Am J Epidemiol 2024; 193:415–425. doi: https://doi.org/10.1093/aje/kwad208 37939072 PMC11484610

[17] Stergiou GS, Alpert BS, Mieke S, Wang J, O’Brien E. Validation protocols for blood pressure measuring devices in the 21st century. J Clin Hypertens (Greenwich) 2018; 20:1096–1099. doi: https://doi.org/10.1111/jch.13294 30003697 PMC8030852

[18] Jones MA, Diesel SJ, Barone Gibbs B, Whitaker KM. Concurrent agreement between ActiGraph and activPAL for measuring physical activity in pregnant women and office workers. J Meas Phys Behav 2022; 5:69–75. doi: https://doi.org/10.1123/jmpb.2021-0050 36340243 PMC9635580

[19] Kozey-Keadle S, Libertine A, Staudenmayer J, Freedson P. The feasibility of reducing and measuring sedentary time among overweight, non-exercising office workers. J Obes 2012; 2012:282303. doi: https://doi.org/10.1155/2012/282303 22175004 PMC3228288

[20] Eguchi K, Kuruvilla S, Ogedegbe G, Gerin W, Schwartz JE, Pickering TG. What is the optimal interval between successive home blood pressure readings using an automated oscillometric device? J Hypertens 2009; 27:1172–1177. doi: https://doi.org/10.1097/hjh.0b013e32832a6e39 19462492 PMC2941726

[21] R Core Team. R: A Language and Environment for Statistical Computing. Published online 2024. https://www.R-project.org/

[22] Pinheiro J, Bates D, R Core Team. nlme: Linear and Nonlinear Mixed Effects Models. Published online 2023. https://CRAN.R-project.org/package=nlme

[23] Canoy D, Nazarzadeh M, Copland E, Bidel Z, Rao S, Li Y, Rahimi K. How much lowering of blood pressure is required to prevent cardiovascular disease in patients with and without previous cardiovascular disease? Curr Cardiol Rep 2022; 24:851–860. doi: https://doi.org/10.1007/s11886-022-01706-4 35524880 PMC9288358

[24] Williams B, Mancia G, Spiering W, Agabiti Rosei E, Azizi M, Burnier M, Clement DL, Coca A, de Simone G, Dominiczak A, Kahan T, Mahfoud F, Redon J, Ruilope L, Zanchetti A, Kerins M, Kjeldsen SE, Kreutz R, Laurent S, Lip GYH, McManus R, Narkiewicz K, Ruschitzka F, Schmieder RE, Shlyakhto E, Tsioufis C, Aboyans V, Desormais I; ESC Scientific Document Group . 2018 ESC/ESH Guidelines for the management of arterial hypertension: the Task Force for the management of arterial hypertension of the European Society of Cardiology (ESC) and the European Society of Hypertension (ESH). Eur Heart J 2018; 39:3021–3104. doi: https://doi.org/10.1093/eurheartj/ehy339 30165516

[25] Imai Y, Kario K, Shimada K, Kawano Y, Hasebe N, Matsuura H, Tsuchihashi T, Ohkubo T, Kuwajima I, Miyakawa M; Japanese Society of Hypertension Committee for Guidelines for Self-monitoring of Blood Pressure at Home. The Japanese Society of Hypertension Guidelines For Self-Monitoring Of Blood Pressure At Home (Second Edition). Hypertens Res 2012; 35:777–795. doi: https://doi.org/10.1038/hr.2012.56 22863910

[26] Groenland EH, Bots ML, Visseren FLJ, McManus RJ, Spiering W. Number of measurement days needed for obtaining a reliable estimate of home blood pressure and hypertension status. Blood Press 2022; 31:100–108. doi: https://doi.org/10.1080/08037051.2022.2071674 35574599

[27] Barone Gibbs B, Muldoon MF, Conroy MB, Paley JL, Shimbo D, Perera S. Influence of recent standing, moving, or sitting on daytime ambulatory blood pressure. J Am Heart Assoc 2023; 12:e029999. doi: https://doi.org/10.1161/JAHA.123.029999 37589152 PMC10547321

[28] Brohan MP, Daly FP, Kelly L, McCarthy FP, Khashan AS, Kublickiene K, Barrett PM. Hypertensive disorders of pregnancy and long-term risk of maternal stroke—a systematic review and meta-analysis. Am J Obstet Gynecol 2023; 229:248–268. doi: https://doi.org/10.1016/j.ajog.2023.03.034 36990309

[29] Cunningham MW, LaMarca B. Risk of cardiovascular disease, end-stage renal disease, and stroke in postpartum women and their fetuses after a hypertensive pregnancy. Am J Physiol Regul Integr Comp Physiol 2018; 315:R521–R528. doi: https://doi.org/10.1152/ajpregu.00218.2017 29897824 PMC6172627

[30] Kitt JA, Fox RL, Cairns AE, Mollison J, Burchert HH, Kenworthy Y, McCourt A, Suriano K, Lewandowski AJ, Mackillop L, Tucker KL, McManus RJ, Leeson P. Short-term postpartum blood pressure self-management and long-term blood pressure control: a randomized controlled trial. Hypertension 2021; 78:469–479. doi: https://doi.org/10.1161/HYPERTENSIONAHA.120.17101 34176288 PMC8260340

[31] Gamble DT, Brikinns B, Myint PK, Bhattacharya S. Hypertensive disorders of pregnancy and subsequent cardiovascular disease: current national and international guidelines and the need for future research. Front Cardiovasc Med 2019; 6:55. doi: https://doi.org/10.3389/fcvm.2019.00055 31157237 PMC6533460

